# Lysophosphatidic Acid Alters The Expression of Apoptosis Related Genes and miR-22 in Cultured and Autotransplanted Ovaries

**DOI:** 10.22074/cellj.2021.7303

**Published:** 2021-10-30

**Authors:** Maryam Dehghan, Shirin Shahbazi, Mojdeh Salehnia

**Affiliations:** 1 Department of Anatomy, Faculty of Medical Sciences, Tarbiat Modares University, Tehran, Iran; 2 Department of Medical Genetics, Faculty of Medical Sciences, Tarbiat Modares University, Tehran, Iran

**Keywords:** Apoptosis, Autotransplantation, BAX Protein, Lysophosphatidic Acid, Ovarian Follicle

## Abstract

**Objective:**

The aim of this study was to evaluate the effect of lysophosphatidic acid (LPA) on the follicular development, incidence of cell death, and expressions of apoptosis related genes and miR-22 in transplanted ovaries.

**Materials and Methods:**

In this experimental study, three-week-old mice ovaries were cultured for 24 hours in the presence and absence of LPA, and we assessed cell survival and normal follicular rates in some of the cultured ovaries. The remaining cultured ovaries were autotransplanted in the presence and absence of LPA as four experimental groups (LPA-/LPA-, LPA-/LPA+, LPA+/LPA-, LPA+/LPA+). The follicular development, immunohistochemistry for BAX, and expressions of genes related to apoptosis and miR-22 by real time reverse transcription polymerase chain reaction (RT- PCR) were studied at the first oestrous cycles in the recovered ovaries. Sera 17-β-oestradiol (E2) and progesterone (P4) levels were also assessed.

**Results:**

Both cell survival and normal follicular rates were significantly higher in cultured ovaries in the presence of LPA after 24 hours (P<0.05). There was an increase in follicular development in comparison with the intact control group in the four transplanted groups (P<0.05). The LPA+/LPA-group had significantly higher follicular development, a decline in BAX positive cells, and a decrease in pro-apoptotic gene expressions in parallel with enhanced expression of anti-apoptotic and miR-22 genes and higher levels of hormones compared with the non-treated and intact control groups (P<0.05).

**Conclusion:**

LPA, as a survival factor, improves follicular development in transplanted ovaries by providing a balance between the anti- and pro-apoptotic genes in association with an increase in miR-22 expression.

## Introduction

The follicular apoptosis was tack placed in grafted ovaries and it reduced the number of follicles within the transplanted tissue. The ovarian apoptosis is mediated by two main internal and external pathways that are involved by some regulatory proteins such as BAX, BAD as pro-apoptotic and BCL2, as an anti-apoptotic protein (1).

MicroRNAs are small non-coding RNAs that regulate gene expressions and inhibit messenger RNA translation. They have an important role in controlling apoptosis in several cell types (2-10).

Among the microRNAs, *miR-22* plays an essential role in apoptosis inhibition in several cell types; however, its involvement in regulation of ovarian follicular apoptosis is not well-known (8-16). Real-time reverse transcription polymerase chain reaction (RT-PCR) assessment of *miR-22* expression in healthy and atretic follicles showed that its expression decreased during mouse follicular atresia. In addition, it was suggested that *miR-22* suppressed mouse granulosa cell apoptosis and decreased *Bax* expression in these cells *in vitro* (12).

Apoptosis could be induced by physical conditions such as oxidative stress after transplantation of ovarian tissues (17). Therefore, more attention has been focused on the use of antioxidants, growth factors, and anti-apoptotic factors to improve the quality of ovarian grafts (18-20). Lysophosphatidic acid (LPA) is a small molecule (430-480 Da) that has been detected in several tissues and biological fluids such as serum, follicular fluid, and plasma. LPA is produced from the phospholipids of the cellular membrane by two enzymes, autotaxin and phospholipase A. Ovarian cells, endometrial cells, mast cells, erythrocytes and neurons produce LPA and it has physiological as well as pathological functions in these cells (21). LPA regulates anti-apoptotic, differentiation and proliferation processes via its G protein-coupled receptors (LPA1-6) on granulosa cells (22-24). LPA has been shown to enhance oocyte maturation and follicular development in bovine, mouse and human (25-31). Abedpour et al. (29, 31) reported that supplementation of mouse ovary culture media with LPA for seven days enhanced the developmental rate of follicles and LPA acted as an anti-cell death factor. A similar effect was reported by Boruszewska et al. (25), when they added LPA to bovine oocyte maturation media. Despite the increased interest in improving ovarian follicle survival after transplantation, to the best of our knowledge no report has assessed the effects of LPA treatment on the improvements in follicular development during the pre-transplantation period (*in vitro* culture) and transplantation of mouse ovaries. The aim of this study was to clarify several aspects of LPA supplementation, as an anti-apoptotic factor, during *in vitro* culture and transplantation of mouse ovaries. We investigated the following: i. Cell survival and normal follicular development at the morphological level, ii. The incidence of BAX positive cells by immunohistochemistry and the expressions of apoptosis related genes (*Bax, Bad*, and *Bcl2*), and iii. The level of *miR-22*, as an inhibitory factor of apoptosis, in transplanted ovaries in response to LPA treatment.

## Materials and Methods

All materials were obtained from Sigma-Aldrich (Dusseldorf, Germany) unless otherwise indicated.

### Animals

The Ethics Committee for Animal Research at Tarbiat Modares University (IR.TMU.REC.1395.530) approved this experimental study. Three-week-old female NMRI mice (n=114) comprised the experimental group and six-week-old adult female NMRI mice (n=14) were the control group. The mice were kept under controlled conditions (20-24˚C, 12/12 hour light: dark cycle, and 40- 50% humidity) at the animal house of Tarbiat Modares University, Tehran, Iran. 

### Ovary removal

The animals (n=114) were anesthetized with intraperitoneal injections of ketamine (50 mg/kg) and xylazine (5 mg/kg). The right ovary of each mouse was removed through a dorsal horizontal incision and then cultured *in vitro*. The left ovary was left intact.

### *In vitro* culture of ovarian tissues

The right ovaries (n=114) were individually cultured on inserts (Millicell-CM, 0.4-µm pore size, Millipore Corp, Billerica, MA, US) at 37˚C and 5% CO_2_ for 24 hours in the presence (n=57) and absence (n=57) of LPA. The culture media consisted of 300 µl α-MEM medium supplemented with 5% foetal bovine serum; 1% insulin, transferrin, and selenium (Invitrogen, UK), and 100 mIU/ml recombinant follicle stimulating hormone (Serono, Switzerland). The treated group had 20 µM LPA (INstruchemie, The Netherlands) added to the culture medium (31).

The cultured ovaries were observed under an inverted microscope and some of the cultured ovaries were considered for assessment of ovarian cell survival using Calcein AM (n=6 for the LPA-treated group and n=6 for the non-treated group) and for morphological analysis with haematoxyline and eosin (n=5 for the LPA-treated group and n=5 for the non-treated group). The other cultured ovaries were encapsulated with sodium alginate and then transplanted under kidney capsule (n=45 for the LPA-treated group and n=45 for the non-treated group). 

### Assessment of ovarian cell survival after *in vitro* culture

We evaluated and compared the survival rate of ovarian cells 24 hours after the *in vitro* culture in the presence and absence of LPA. The cultured ovaries (n=6 per group) were incubated with 3 mg/ml collagenase type I at 37˚C, washed with phosphate-buffered saline (PBS), and passed through a filter that had a pore size of 40 μm. The collected cells were stained with double fluorescent labelling dyes with Calcein AM and ethidium homodimer according to a live/dead viability kit (Live/Dead Viability/Cytotoxicity Kit, Molecular Probes, Life Technologies, Germany). Briefly, the cells were washed with PBS and incubated in 1.6 µM Calcein AM and 5.5 µM EthD-1 for 30-45 minutes at 37˚C in the dark. Then, they were placed on slides and covered by a coverslip and observed under a fluorescent microscope. The cells were reported as viable (stained green) or nonviable (stained red). Photographs (n=5) of each sample were prepared and imported into ImageJ software, then the mean percent of viable and nonviable cells were counted per 1000 µm^2^ in each sample.

### Encapsulation of *in vitro* cultured ovaries in sodium alginate

The cultured ovaries were encapsulated in sodium alginate (n=92); briefly, the ovaries were individually immersed into 5 µl droplets of sodium alginate at a concentration of 0.5% (w/v) in PBS (with or without LPA) at room temperature and then they were gently transferred into a cross-linking solution (50 mM CaCl_2_ and 140 mM NaCl) for two minutes (31). In the LPA-treated group, 20 µM LPA was added to the sodium alginate solution. Finally, the encapsulated ovaries from both groups were individually autotransplanted under kidney capsules as follows.

### Ovarian transplantation into the kidney capsule

The ovaries were divided into the following four experimental groups:

Experimental group A: The right ovaries (n=14) were removed, cultured for 24 hours, encapsulated in sodium alginate without any supplementation, and subsequently autotransplanted into the right kidney capsule (LPA^-^ / LPA^-^).

Experimental group B: The right ovaries (n=5) were removed and cultured without LPA for 24 hours, then encapsulated in sodium alginate with LPA and autotransplanted under the right kidney capsule (LPA^-^ / LPA^+^). 

Experimental group C: The right ovaries (n=14) were removed and cultured in medium that contained LPA for 24 hours, encapsulated in sodium alginate without any supplementation, and subsequently autotransplanted into the right kidney capsule (LPA^+^ /LPA^-^). 

Experimental group D: The right ovaries (n=5) were removed and cultured in medium supplemented with LPA for 24 hours, encapsulated in sodium alginate that contained LPA, and autotransplanted into the right kidney capsule (LPA^+^ /LPA^+^). 

The left ovaries of the mice were intact in all of the experimental groups.

Prior to transplantation, the mice from the four experimental groups were anesthetised with i.p. injections of ketamine (50 mg/kg) and xylazine (5 mg/kg). A dorsal horizontal incision was generated and the right kidney capsule was exposed. The cultured encapsulated ovary was inserted under the kidney capsule through a tiny hole by using watchmaker’s forceps. The body wall and skin incision were closed and the mice were kept under aseptic conditions until the healed ovaries were collected.

### Vaginal cytology

Three weeks after the ovaries were transplanted, the stages of the mice oestrous cycles were confirmed daily by vaginal cytology under a light microscope at 400x magnification. The stages of the oestrous cycle were identified as prooestrus, oestrus, metoestrus, or dioestrus by the presence or absence of nucleated epithelial cells, cornified epithelial cells, and leukocytes.

### Hormonal assay

We evaluated the endocrine function of the ovaries in the experimental groups with lower (group A) and higher (group C) follicular developmental rates. For this analysis and prior to collection of the ovaries, we obtained blood samples by cardiac puncture with a needle from the heart without thoracotomy in mice. Then, the sera separated and keep at -20˚C until hormonal analysis (n=3 for each group). The concentrations of 17-β-estradiol (E2) and progesterone (P4) were measured using a microplate enzyme immunoassay kit (Biotest AG, Germany) that had a sensitivity of 6.5 pg/mL. The sera of six-week-old mice, as the intact control group (n=3), were collected and analysed in the same manner as the experimental groups.

### Recovery of transplanted ovaries

The animals were sacrificed by cervical dislocation during the prooestrus phase of the first oestrous cycle. The transplanted ovaries were recovered and collected, first for the morphological study, then for immunohistochemical and molecular analyses for apoptosis and *miR-22* expression.

### Histological evaluation

For morphological analysis, the ovaries cultured *in vitro* (for 24 hours) in the presence and absence of LPA and recovered tissue at first oestrus cycles (5 ovaries in each group) were fixed in Bouin’s solution for 8 hours. They were embedded in paraffin wax, serially sectioned at 5 μm and mounted on slides with 5^th^ intervals and stained with haematoxylin and eosin method. The same procedure was done for ovaries obtained from six-week-old mice during the prooestrus phase, as the intact control group (n=5).

The tissue sections were studied under a light microscope in order to determine the normal and degenerated follicles at different developmental stages. The ovarian follicles were classified as primordial (oocytes surrounded by single layer of squamous pregranulosa cells), primary (surrounded by single layer of cuboidal granulosa cells), preantral (two or more layers of cuboidal granulosa cells), and antral follicles with the antrum cavity (31). In order to avoid counting follicles more than once, we only counted follicles that were in the sections with visible oocyte nuclei.

### Immunohistochemistry

Another set of tissue sections from the experimental group that had lower (group A) and higher (group C) follicular developmental rates were placed on coated slides and used for the immunohistochemical studies. The tissue sections from each sample of the recovered and control ovaries (n=3 ovaries in each group) were randomly deparaffinised, rehydrated in descending ethanol solutions, and finally washed in PBS. Antigen retrieval was performed by boiling the tissue slides in 10 mM citrate buffer (10 mM, pH=6) in a microwave oven for 10 minutes at 700 W. Then, they were cooled at room temperature and washed in PBS. The sections were immersed in 0.3% Triton X100 for 30 minutes then washed in PBS, blocked with goat serum (30 minutes) and incubated overnight at 4˚C in a humid chamber with the primary antibody, anti-BAX polyclonal antibody, (Elabscience Biotechnology Co, Wuhan, China; 1:100). The tissue sections were washed, incubated with polyclonal goat anti-rabbit antibody (Elabscience Biotechnology Co, Wuhan, China; 1:20) conjugated with FITC for 30 minutes, and washed in PBS. Then, the tissue slides were evaluated under a fluorescent microscope at ×400 magnification. Photographs of each section were prepared and imported into ImageJ software. Then, we counted the number of BAX positive cells per 1000 μm^2^ of ovarian tissue in three sections from each sample. 

### RNA extraction 

Experimental groups A and C and the intact control group were considered for molecular analysis (n=6 per group). Total RNA was extracted using TRIzol reagent according to the manufacturer’s instructions in the RNeasy Mini Kit (Qiagen, Germany). Briefly, the ovaries were individually homogenized in 0.5 ml of TRIzol reagent, then 0.2 ml of chloroform was added per one ml of TRIzol and the samples were centrifuged at 12000 g for 10 minutes. The upper colourless aqueous phase was transferred to a fresh 1.5 ml microtube and 500 μl of 100% isopropanol (Sigma-Aldrich, Germany) was added, then the samples were centrifuged at 12000 g for 10 minutes followed by the addition of 1.5 ml 70% ethanol per one ml of TRIzol reagent to the samples. The RNA concentration was determined with a spectrophotometer (Eppendorf, Germany).

### cDNA synthesis

cDNA was synthesized with a cDNA synthesis kit (Thermo Fisher Scientific, Germany) according to the manufacturer’s instructions. Oligo (dT) was used for cDNA synthesis and the reverse transcriptase reaction was incubated at 65˚C for 5 minutes and at 42˚C for 60 minutes. In order to evaluate *miR-22* gene expression, cDNA was synthesized using a commercial cDNA synthesis kit (Thermo Fisher Scientific, Germany). Stem loop was used for cDNA synthesis and the reverse transcriptase reaction was incubated at 16˚C for 30 minutes and at 42˚C for 60 minutes. After inactivation of the reverse transcriptase enzyme at 70˚C for 5 minutes, the product was stored at -20˚C until real-time RT-PCR assessment.

### Real-time reverse transcription polymerase chain reaction 

Primer-BLAST tool in NCBI was used to design primers for the apoptosis related genes that included *Bax, Bcl2*, and *Bad* and the housekeeping gene,* β-actin*, and for *miR-22* and its housekeeping gene (*U6*) (Table S1, See supplementary Online Information at www. celljournal.org). Real-time RT-PCR was performed using a QuantiTect SYBR Green RT-PCR Kit (Qiagen, Germany). The thermal program of real-time RT-PCR was set with the following parameters: initial holding stage for 5 minutes at 95˚C; 40 cycles with cycling stages of 15 seconds at 95˚C, 58˚C for 30 seconds, and 72˚C for 15 seconds; and a melting curve stage at 95˚C for 15 seconds, 60˚C for 1 minute, and 95˚C for 15 seconds. After completing the PCR run, the melting curve was analysed using the amplicon. 

### Statistical analysis 

All experiments were conducted with a minimum of three replicates. Statistical analysis was performed using SPSS software (SPSS Inc., Chicago, USA). Values are written as mean ± SD. One-way ANOVA and the post hoc Tukey test were used to compare differences in follicular count, BAX positive cells, mRNA expression, and hormone levels. A P<0.05 was considered to be statistically significant.

## Results

### Morphology of cultured ovaries under an inverted microscope

**Fig.1:**
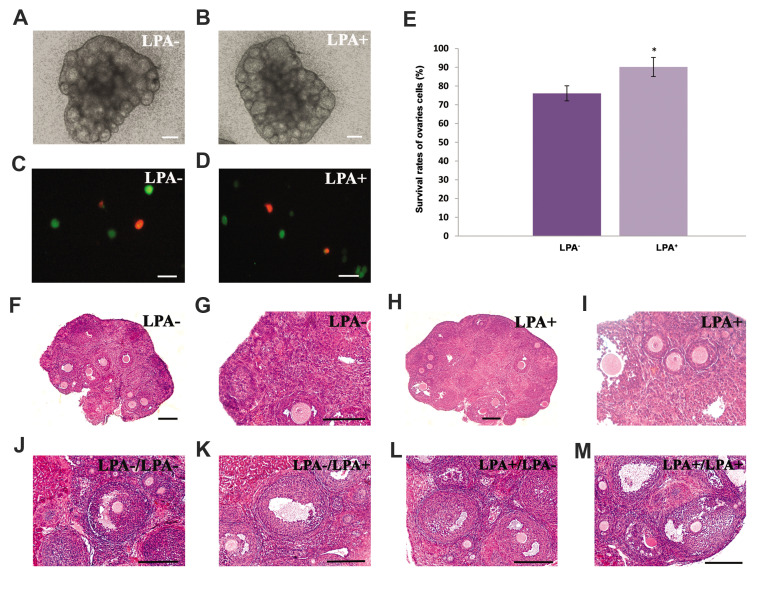
Phase contrast micrograph of cultured ovaries in the studied groups. **A.** Cultured without any supplementation for 24 hours (LPA^-^) and **B.** Cultured in the presence of LPA for 24 hours (LPA^+^). **C.** Representative fluorescence microscopy images of isolated cells derived from cultured ovaries that were stained with Calcein AM in the absence of LPA and **D.** The LPA-treated group. The viable cells stained green and the nonviable cells stained red. **E.** Comparison of the survival rates of ovarian isolated cells in the studied groups. *; Significant difference with other group (P<0.05). **F, G.** Light microscopic observation of haematoxylin and eosin stained tissue sections of the ovaries cultured for 24 hours in LPA^-^ group and **H, I.** In LPA^+^ group. Representative photographs of transplanted ovarian sections in the experimental groups are shown in the last row. **J. **Experimental group A (LPA-/LPA). **K. **Experimental group B (LPA^-^ / LPA^+^).** L. **Experimental group C (LPA^+^ /LPA^-^). **M.** Experimental group C (LPA^+^ /LPA^+^). These assessments were done in five repeats in the studied groups. LPA; Lysophosphatidic acid (scale bar: A, B: 300 μm, C, D: 50 μm, F, H, G, I, J, K, L, M: 100 μm).

Figure 1A, B shows the morphological characteristics of the cultured ovaries in the presence and absence of LPA under an inverted microscope. The follicles grew within the cortical parts of the ovaries and the antral follicles had a spherical shape with large, clear antral cavities.

### The survival rate of cultured ovarian cells

Figure 1C, D shows images of a Calcein AM stained cell suspension obtained from the ovaries cultured *in vitro* for 24 hours in the presence and absence of LPA. The images in the figure show that the viable cells stained green whereas nonviable cells stained red. A significantly greater (P<0.05) mean percentage of cells survived in the LPA-treated group (90.17 ± 5.06%) compared to the non-treated group (76.08 ± 4.01%, Fig .1E). 

### Morphological analysis of *in vitro* cultured ovaries

Representative light microscopy photograph of sections from the *in vitro* cultured ovaries that were stained with haematoxyline and eosin after 24 hours are shown in Figure 1F-G. Follicles at different developmental stages had normal structures in both of the studied groups. 

From the 482 total follicles counted in the non-treated group, 99.21 ± 0.06% had normal morphology and out of the 534 total follicles in the LPA-treated group, 99.69 ± 0.07% had normal morphology. This rate was significantly higher in the LPA-treated group in comparison with the non-treated group (P<0.05). There was no significant difference between these groups in terms of percentages of follicles at the different developmental stages (Table 1).

### Morphological analysis in recovered transplanted ovaries at the first oestrous cycle

The morphology of transplanted ovaries sections in four experimental groups and the intact control group at the proestrus phase is shown in Figure 1J-M. Follicles from both study groups, which are grown at different developmental stages, are visible in this figure. The total number of follicles counted were 539 in the intact control group. The total number of counted follicles in the experimental groups were 415 (group A), 489 (group B), 525 (group C), and 529 (group D).

**Table 1:** The percentages of normal follicles at different developmental stages in cultured mouse ovaries


Group	Total no. F	Normal F.	Degenerated F.	Primordial F.	Primary F.	Preantral F.

LPA^-^	482	478 (99.21 ± 0.06)	4 (0.79 ± 0.06)	239 (49.91 ± 1.18)	112 (23.50 ± 0.56)	127 (26.59 ± 0.65)
LPA^+^	534	532 (99.69 ± 0.07)^a^	2 (0.31 ± 0.07)^a^	262 (49.22 ± 1.16)	126 (23.72 ± 0.56)	144 (27.06 ± 0.63)


Data are presented as n (mean ± SD%). The percentages of follicles at different developmental stages in all studied groups were calculated according to the normal follicles. These assessments were done in five repeats in the studied groups. LPA-; Untreated, LPA+; Treated with LPA, LPA; Lysophosphatidic acid, F; Follicles, and a; Significant differences with the LPA- group (P<0.05).

There was no significant difference in terms of the mean percentages of normal follicles between the intact control group (99.76 ± 0.07%) and experimental groups C (99.68 ± 0.08%) and D (99.70 ± 0.08%) (Table 2, P>0.05). The mean percentages of primordial and primary follicles significantly decreased and the preantral and antral follicles increased in all four experimental groups in comparison with the intact control group (P<0.05). Among the experimental groups, the lowest percentage of primordial follicles and the highest percentage of antral follicles were seen in experimental group C (P<0.05).

### Immunohistochemistry for BAX 

**Fig.2:**
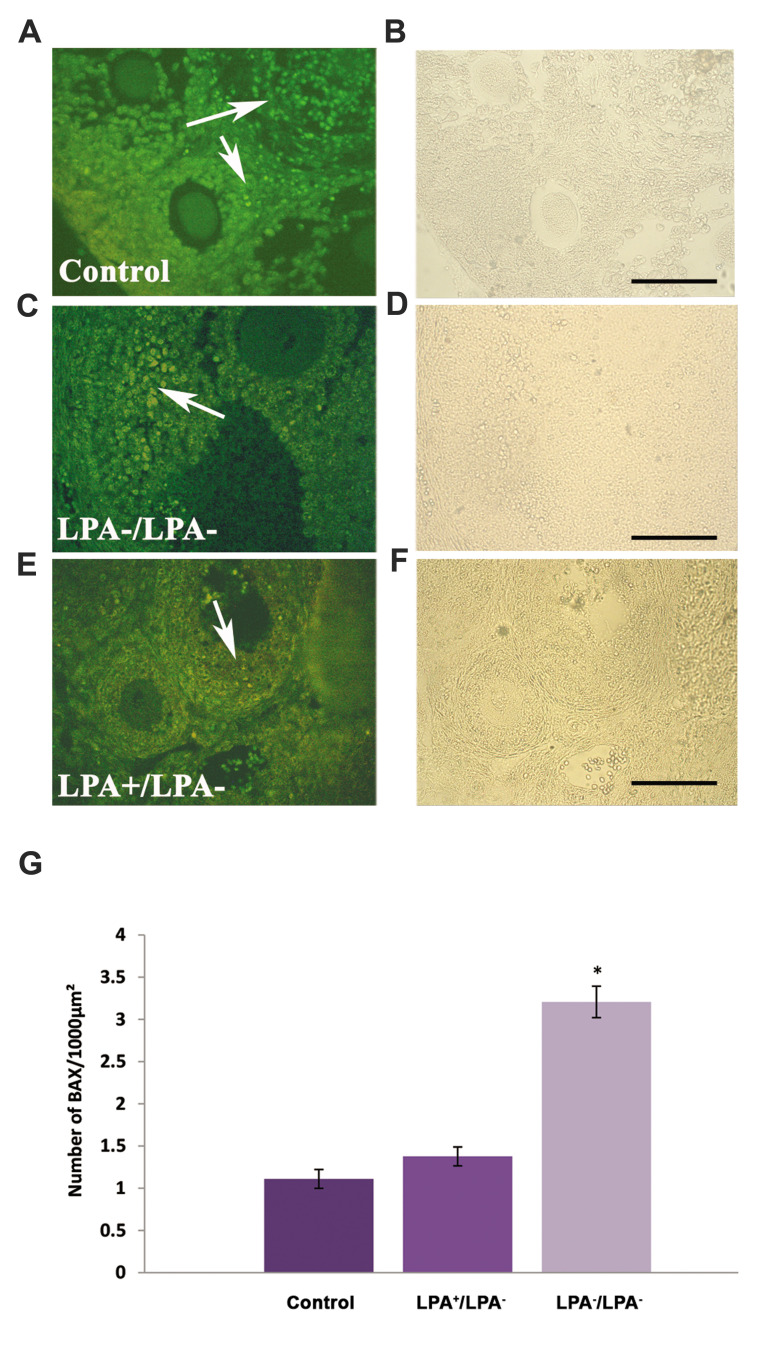
Photomicrographs of transplanted mouse ovarian sections immunostained for BAX antibody and observed under fluorescent microscopy (first column) and by phase contrast microscopy (second column). **A., a. **Intact control group, **B., b.** Experimental group A (LPA^-^ /LPA^-^), **C., c.** Experimental group C (LPA^+^ /LPA^- ^). Green colour shows the positive cell reaction (white arrow) for the BAX antibody, and **D.** A comparison of the number of BAX positive cells/1000 µm^2^ in the studied groups. *; Significant difference with the other groups (P<0.05). The immunocytochemistry analysis was repeated three times. LPA; Lysophosphatidic acid (scale bar: 100 µm).

Figure 2A-C shows photomicrographs related to the immunohistochemistry of the recovered ovarian sections in experimental groups A and C, and the intact control ovaries. The number of BAX positive cells (green colour, Fig .2) was significantly higher in experimental group A (3.20 ± 0.18 per 1000 µm^2^) in comparison with the intact control (1.11 ± 0.11 per 1000 µm^2^) and experimental group C (1.37 ± 0.11 per 1000 µm^2^, P<0.05). 

### Real-time reverse transcription polymerase chain reaction

**Fig.3:**
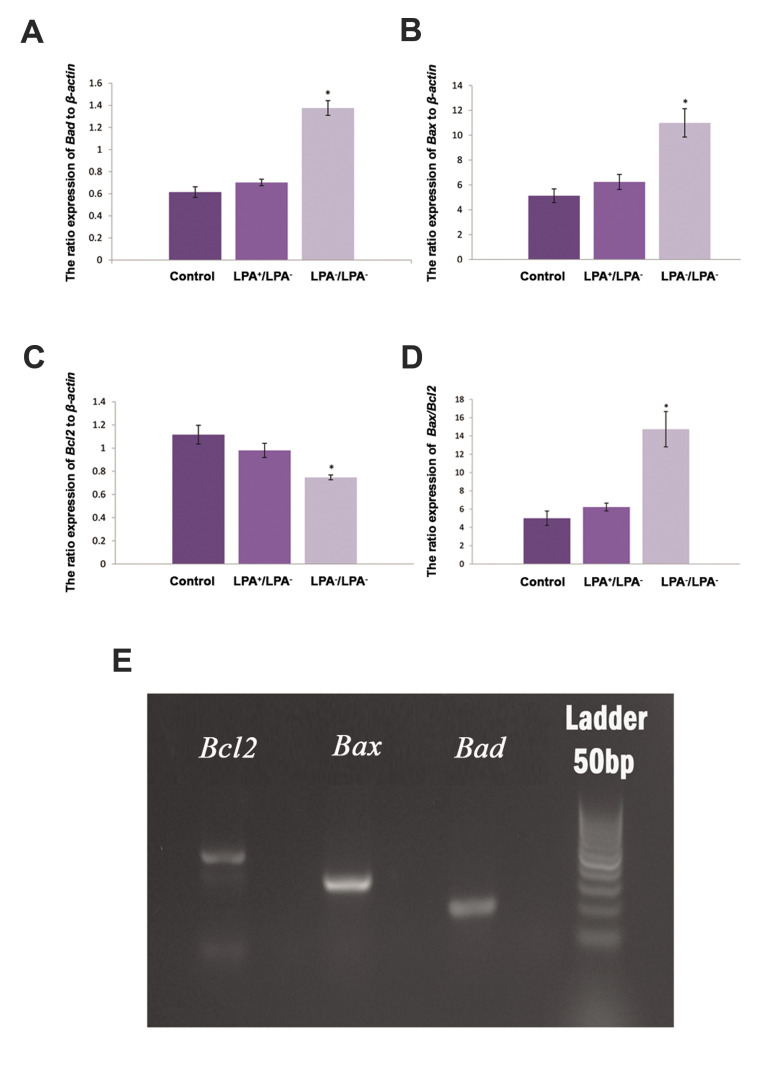
The relative expression ratio of pro- and anti-apoptotic genes to* β-actin* in studied groups. The comparison of gene expression in transplanted mouse ovaries in experimental groups A (LPA^-^ /LPA^-^) and C (LPA^+^ /LPA^-^), and the intact control group are shown in parts **A. ***Bad*, **B. ***Bax*, **C. ***Bcl2*, and **D. ***Bax/Bcl2*. **E.** Agarose gel electrophoresis of the PCR products of the genes related to apoptosis. Lane 1; *Bad*, Lane 2; *Bax*, Lane 3; *Bcl2*, and Lane 4; 50 bp ladder. These assessments were done in three repeats in the studied groups. LPA; Lysophosphatidic acid and *; Significant difference with the other groups (P<0.05), and PCR; Polymerase chain reaction.

Figure 3 shows the relative expression ratios of the apoptosis related genes to* β-actin*. In the intact control group, the expression ratios were 5.128 ± 0.55 for *Bax*, 0.615 ± 0.04 for *Bad* and 1.116 ± 0.08 for *Bcl2*. In experimental group A, they were 10.99 ± 1.14 (*Bax*), 1.376 ± 0.06 (*Bad*), and 0.747 ± 0.20 (*Bcl2*) and for experimental group C, the expression ratios were 6.239 ± 0.60 (*Bax*), 0.702 ± 0.02 (*Bad*), and 0.980 ± 0.06 (*Bcl2*). There were significant differences between experimental group A and the two other groups in terms of the gene expressions (P<0.05). 

**Fig.4:**
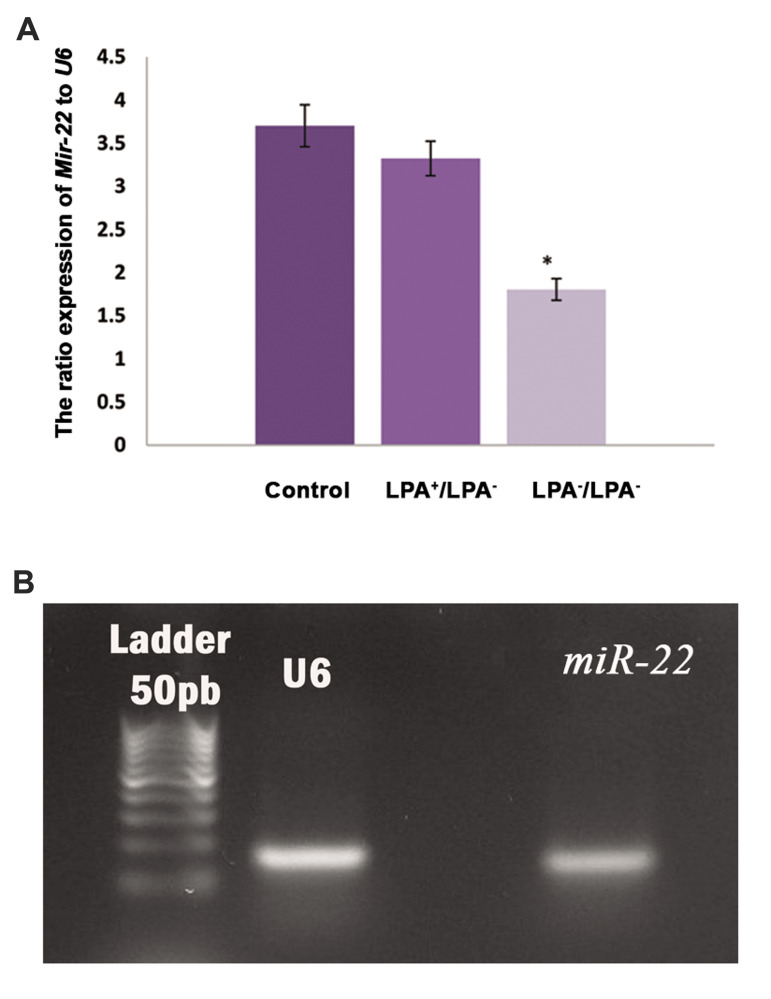
The relative expression ratio of the *miR-22* gene to U6 in studied groups. **A. **The comparison of gene expression in transplanted mouse ovaries in experimental groups A (LPA^-^ /LPA^-^) and C (LPA^+^ /LPA^-^), and the intact control group. **B.** Agarose gel electrophoresis of the PCR product of the *miR-22* gene. Lane 1; 50 bp ladder, Lane 2; U6, and Lane 3; *miR-22*. These assessments were done in three repeats in the studied groups. LPA; Lysophosphatidic acid, *; Significant difference with the other groups (P<0.05), and PCR; Polymerase chain reaction.

The relative expression ratio of *miR-22* to* U6* in the intact control group was 3.702 ± 0.24, in experimental group A it was 1.804 ± 0.12 and in experimental group C it was 3.323 ± 0.20. There was a higher expression ratio of *miR22* in experimental group C in comparison with the two other groups (P<0.05, Fig .4).

**Table 2:** The percentages of normal follicles at different developmental stages in the first oestrous cycle


Group	Total no. F.	Normal F.	Primordial F.	Primary F.	Preantral F.	Antral F.

Intact control(Six-week-old ovaries)	539	538 (99.76 ± 0.07)	280 (52.04 ± 0.60)	118 (21.93 ± 0.68)	117 (21.74 ± 0.63)	23 (4.29 ± 0.61)
Exp. A (LPA^-^/LPA^-^)	415	412 (99.27 ± 0.05)^a^	113 (27.38 ± 0.74)^a^	75 (18.17 ± 0.58)^a^	145 (35.24 ± 0.77)^a^	79 (19.21 ± 0.59)^a^
Exp. B (LPA^-^/LPA^+^)	489	487 (99.51 ± 0.08)^ab^	70 (14.33 ± 0.75)^ab^	58 (11.89 ± 0.63)^ab^	256 (52.65 ± 1.15)^ab^	103 (21.13 ± 0.56)^ab^
Exp. C (LPA^+^/LPA^-^)	525	523 (99.68 ± 0.08)^b^	53 (10.09 ± 0.72)^ab^	43 (8.24 ± 0.61)^ab^	289 (55.27 ± 0.74)^ab^	138 (26.40 ± 0.61)^ab^
Exp. D (LPA^+^/LPA^+^)	529	527 (99.70 ± 0.08)^bc^	70 (13.34 ± 0.80)^abcd^	50 (9.44 ± 0.63)^abcd^	284 (53.88 ± 0.88)^abcd^	123 (23.34 ± 0.55)^abcd^


Data are presented as n (mean ± SD%). The percentages of follicles at different developmental stages in all studied groups were calculated according to the normal follicles. These assessments were done in five repeats in the studied groups. LPA^-^ ; Untreated, LPA^+^ ; Treated with LPA, LPA; Lysophosphatidic acid, F; Follicles, ^a^ ; Significant differences with intact control group (P<0.05), ^b^ ; Significant differences with the LPA^-^ /LPA^-^group (P<0.05),^ c^ ; Significant differences with the LPA^-^ /LPA^+^ group (P<0.05), and ^d^ ; Significant differences with the LPA^+^ / LPA^-^group (P<0.05).

### 17-β-estradiol and progesterone levels

The concentrations of E2 and P4 in sera of the intact control and experimental groups A and C are presented and compared in Table S2 (See supplementary Online Information at www.celljournal.org). Experimental group C had significantly higher levels of E2 and P4 compared to the intact control and experimental group A (P<0.05).

## Discussion

In this study, in order to improve the quality of the transplanted ovaries, we first examined the effects of LPA supplementation during an *in vitro* culture and tissue transplantation. The results showed that the rate of follicular development in the LPA-treated groups were significantly higher than the non-treated and intact control groups. Follicular development is related to growth and proliferation of follicular cells and to maturation of these cells and oocytes. A possible relation to these results is that LPA might act as a growth factor to stimulate the proliferation of follicular granulosa and theca cells; therefore, the expansion of follicular cells could shift the growth of primordial and primary follicles to the preantral and antral stages. The action of LPA is mediated directly by binding to its receptors on follicular cells (21, 23). Expressions of LPA receptors were detected in mouse and bovine ovaries (28, 29). Similarly, the *in vitro* expansion of bovine cumulus cells was also demonstrated in response to LPA (25). Sinderewicz et al. (28) postulated that LPA enhanced the growth and development of bovine follicles via expression of LPA receptors and autotaxin genes. On the other hand, binding of LPA to its surface receptors could increase the expression of LPA receptor genes and, in turn, have a positive feedback on the effects of LPA. In this regard, our group has recently reported significant enhancement in the expression of LPAR1-4 receptor genes after LPA was added to the culture media of mouse ovarian tissues (29). 

LPA may also act as a maturation factor. This effect of LPA on oocyte maturation was shown in several studies (25-32). Komatsu et al. (32) suggested that LPA treatment promoted the nuclear maturation of mouse oocytes during IVM through lowering intra-oocyte cAMP levels. Jo et al. (30) demonstrated that addition of LPA to culture media, especially at 30 μM, improved oocyte maturation, fertilization and blastocyst formation in mice. These researchers also reported that LPA stimulated phospholipase C through the G protein on the surface of cumulus cells and activated mitogen-activated protein (MAP) kinase pathways. Addition of LPA to culture media appears to increase oocyte mRNA amount that is considered as a quality markers of oocyte. Also LPA enhances oocyte maturation rates by stimulating the expression of developmental competence-related factors (25, 33). Zhang et al. (27) showed that LPA promoted meiotic progression of porcine oocytes from the germinal vesicle to metaphase II by stimulating the expression of cyclin B1, a marker of cytoplasmic maturation, by activation of the MAP kinase pathway. In addition, it was suggested that LPA might indirectly interact with other factors that stimulate and regulate follicular development. However, this suggestion should be confirmed by more analysis. 

However, we also observed significant enhancement in the amount of E2 and P4 in the LPA-treated group. This result revealed that LPA positively influenced the function of granulosa and theca cells by production of high concentrations of steroid hormones. Similarly, Boruszewska et al. (34) demonstrated that LPA stimulated the synthesis of E2 in bovine granulosa cells by converting the androgens to E2 via cytochrome P450 aromatase in granulosa cells. It has been suggested that LPA participates in ovarian follicle growth and differentiation by stimulation of E2 production, which may occur via an increase in the expression of follicle stimulating hormone receptor. LPA may be involved in autocrine and/ or paracrine signalling between oocyte and cumulus cells during follicular development (23). 

We compared the follicular proportions at different developmental stages at the first oestrus cycle in all transplanted groups to the intact control group. Our results showed the percentage of large antral follicles in all grafted ovaries was significantly higher than the intact control group. This phenomenon showed rapid development and early discharge of ovarian reserve in the experimental groups, especially in experimental group C (LPA^+^ /LPA^-^), which could affect longevity of the transplanted tissue. Possibly, in the transplanted groups, an immediate lack of negative feedback after grafting could facilitate the production of gonadotropin releasing hormone (GnRH), follicle-stimulating hormone (FSH), and luteinizing hormone (LH), which improved follicle growth in the grafted ovaries. This suggestion correlated with premature follicular discharge observed in the transplanted groups in comparison with the intact control group.

Despite follicular loss after ovarian transplantation in terms of ischemia, we noted that the integrity of the tissue was well-preserved in the LPA-treated groups, which was shown by the high survival rate observed with Calcein AM staining, a high percent of normal follicles, and low incidence of BAX positive cells in the LPA-treated group. These observations suggest that LPA acts as a survival factor during *in vitro* culture and encapsulation of tissue within sodium alginate. Hu et al. (35) have reported that LPA is a survival and growth factor, which prevents spontaneous apoptosis through LPA receptor activation of the anti-apoptotic protein AKT/PKB. McLaughlin et al. (36) have indicated that the Akt pathway promotes cell survival by inhibiting pro-apoptotic proteins such as BAD, BAX, forkhead, and p53 and by activating prosurvival proteins such as BCL2. A similar effect of LPA is well-known in other cell types and the results of these studies show that LPA, via its receptors, leads to cell responses that include protection from apoptosis (22-24). At the molecular level, our results confirmed that LPA supplementation of culture media had a positive effect on the decline in transcription levels of pro-apoptotic (*Bax* and *Bad*) genes and an increase in anti-apoptotic (*Bcl2*) gene expression. The *Bax/Bcl2* ratio was lower in ovaries cultured in the presence of LPA compared to the non-treated group. The ratio of *Bcl2* to *Bax* may be an indicator of the tendency of ovarian cells and follicles toward survival or apoptosis (1). BAX, BAD and BCL2 are regulatory proteins that control the mitochondrial pathway of apoptosis; therefore, follicular development competence in the LPA-treated group might be enhanced through modulation of these apoptotic related gene expressions. This conclusion agreed with the findings reported in other cell types or oocytes (26, 33). In these studies, LPA played a role in cell survival by preventing apoptosis through activation of the anti-apoptotic protein or by alterations in the anti-apoptotic and pro-apoptotic balance, which resulted in a significantly lower *Bcl2/Bax* ratio (33). Zhou et al. (37) reported that the anti-apoptotic effects of LPA involve inhibition of caspase and *Bax*, and the activation of *Bcl2*. Treatment of porcine embryos with LPA resulted in increased expression of the anti-apoptotic *Bcl2* gene and decreased expression of the pro-apoptotic *Bax* and *caspase 3* genes (27). Torres et al. (38) showed that addition of LPA to the culture media of bovine embryos reduced *Bax* expression.

In present study, for the first time, we investigated the expression of *miR-22* in correlation with apoptotic related genes and follicular development in cultured and transplanted ovaries. Our results revealed that the higher expression of *miR-22* was associated with a decline in the incidence of BAX positive cells and pro-apoptotic gene expressions in the LPA-treated group. The results of several studies revealed that *miR-22* played an essential role in regulation of apoptosis in different cell types (12- 14). Overexpression of *miR-22* inhibited cardiac myocyte apoptosis (39) and had a neuroprotective effect through reduction in caspase activation (40). *miR-22* might have a similar effect on other cell types, including ovarian cells and follicles. Xiong et al. demonstrated that *miR-22* expression decreased during mouse follicular atresia and they suggested that *miR-22* suppressed mouse granulosa cell apoptosis *in vitro* (12). Lv et al. (14) reported that *miR-22* regulated cell proliferation and inhibited cell apoptosis by targeting the *eIF4EBP3* gene in human cervical squamous carcinoma cells. *miR-22* decreased BAX expression in granulosa cells by targeting the silent mating-type information regulation 2 homologue 1 (*SIRT1*) gene (12). 

## Conclusion

LPA, as a survival factor, improves follicular development in transplanted ovaries by providing a balance between anti-apoptotic and pro-apoptotic genes in association with an increase in *miR-22* expression.

## Supplementary PDF


