# Supplementary material for: Lysophosphatidic Acid Alters The Expression of Apoptosis Related Genes and miR-22 in Cultured and Autotransplanted Ovaries
Source: Cell J. 2021 Oct 30;23(5):584–92. doi: 10.22074/cellj.2021.7303 (PMC8588818; doi:10.22074/cellj.2021.7303)
Supplement: Supplementary file 1 [file Cell-J-23-584-s01.pdf]

www.celljournal.org

Supplementary Information for

Lysophosphatidic Acid Alters The Expression of Apoptosis Related Genes and miR-22 in Cultured and Autotransplanted Ovaries

Maryam Dehghan, Ph.D.[1], Shirin Shahbazi, Ph.D.[2], Mojdeh Salehnia, Ph.D.[1*]

1. Department of Anatomy, Faculty of Medical Sciences, Tarbiat Modares University, Tehran, Iran
2. Department of Medical Genetics, Faculty of Medical Sciences, Tarbiat Modares University, Tehran, Iran

**Corresponding Address: P.O.Box: 14115-111, Department of Anatomy, Faculty of Medical Sciences, Tarbiat Modares University, Tehran, Iran*
Email: salehnim@modares.ac.ir

Table S1: Sequences of the designed primers for the apoptosis related genes and the *miR-22* gene

Gene	Primer sequence (5´-3´)	Accession number	PCR product size (bp)
Bax	F:GCAAGTAGAAGAGGGCAACC	NM-007527.3	160
	R:CGGCGAAATGGAGATGAACTG		
Bcl2	F:GGTGTTCAGATGTCGGTTCA	NM-177410	237
	R:CGTGGTGACTTCGCAGAG		
Bad	F:CGCTTAGAACTGGAGGGAGGA	NM-001285453.1	98
	R:CACTCGGCTCAAACTCTGGG		
β-actin	F: AGTCATAGTCCGCCTAGAAGC	XM-021163894.1	168
	R: TGAAGATCAAGATCATTGCTCCC		
miR-22	F: AGTACGGAAGCTGCCAGTTGAAG		
	R: CAGTGCAGGGTCCGAGGTATTC		
	Stem loop: GTCGTATCCAGTGCAGGGTCCGAGGTATTCGCACTGGATACGACACAGTT		
U6	F: CGCAAGGATGACACGCAAATTC		
	R: CAGTGCAGGGTCCGAGGTATTC		
	Stem loop: GTCGTATCCAGTGCAGGGTCCGAGGTATTCGCACTGGATACGACAAAAATATGG		

PCR; Polymerase chain reaction.

Table S2: The concentration of 17-β-estradiol (E2) and progesterone (P4) in the first oestrous cycle

Groups	E2 (pg/ml)	P4 (ng/ml)
Intact control	87.76 ± 2.13	0.51 ± 0.005
Exp. A (LPA^-/LPA^-)	91.90 ± 3.71	0.63 ± 0.07
Exp. D (LPA^+/LPA^-)	117.46 ± 15.32[ab]	0.93 ± 0.07[ab]

Data are presented as mean ± SD. E2; 17-β-estradiol, P4; Progesterone, [a]; Significant differences with the intact control group (P<0.05), and [b]; Significant differences with the LPA-/LPA- group (P<0.05). These assessments were done in triplicate in the studied groups.
